# Supplementary material for: ARID1A safeguards the canalization of the cell fate decision during osteoclastogenesis
Source: Nat Commun. 2024 Jul 17;15:5994. doi: 10.1038/s41467-024-50225-z (PMC11252270; doi:10.1038/s41467-024-50225-z)
Supplement: Supplementary file 1 — Supplementary Information [file 41467_2024_50225_MOESM1_ESM.pdf]

ARID1A safeguards the canalization of the cell fate decision during osteoclastogenesis

Supplementary Fig. 1 *Arid1a* knockout strategy and efficiency in *LysM-Cre;Arid1a$^{fl/fl}$* mice.

Supplementary Fig. 2. Loss of *Arid1a* in myeloid lineage leads to excessive bone mass.

Supplementary Fig. 3 The proportion and KEGG enrichment analysis of each cell cluster based on the scRNA-seq data.

Supplementary Fig. 4 The constituently expressed *Nfatc1* partially rescues the defective OC differentiation after loss of *Arid1a*.

Supplementary Fig. 5 Expression change of fate decision marker *Cited2* and *Rab38* after loss of *Arid1a*.

Supplementary Fig. 6 Comparison between the binding profiles of ARID1A and BRD4 during osteoclastogenesis.

Supplementary Fig. 7 Expression change of BRD4 and PU.1 after loss of *Arid1a*.

Supplementary Fig. 8 The motif location of PU.1, NFKB1, FOS::JUN and NFATc1 in the *Nfatc1 SE* region.

Supplementary Fig. 9 Gene set enrichment analysis (GSEA) analysis of DEGs in *LysM-Cre;Arid1a$^{fl/fl}$* group compared with control group based on bulk RNA-seq.

Supplementary Fig. 10 Change of BRD9 in RANKL-induced BMCs after loss of *Arid1a*.

Supplementary Fig. 1

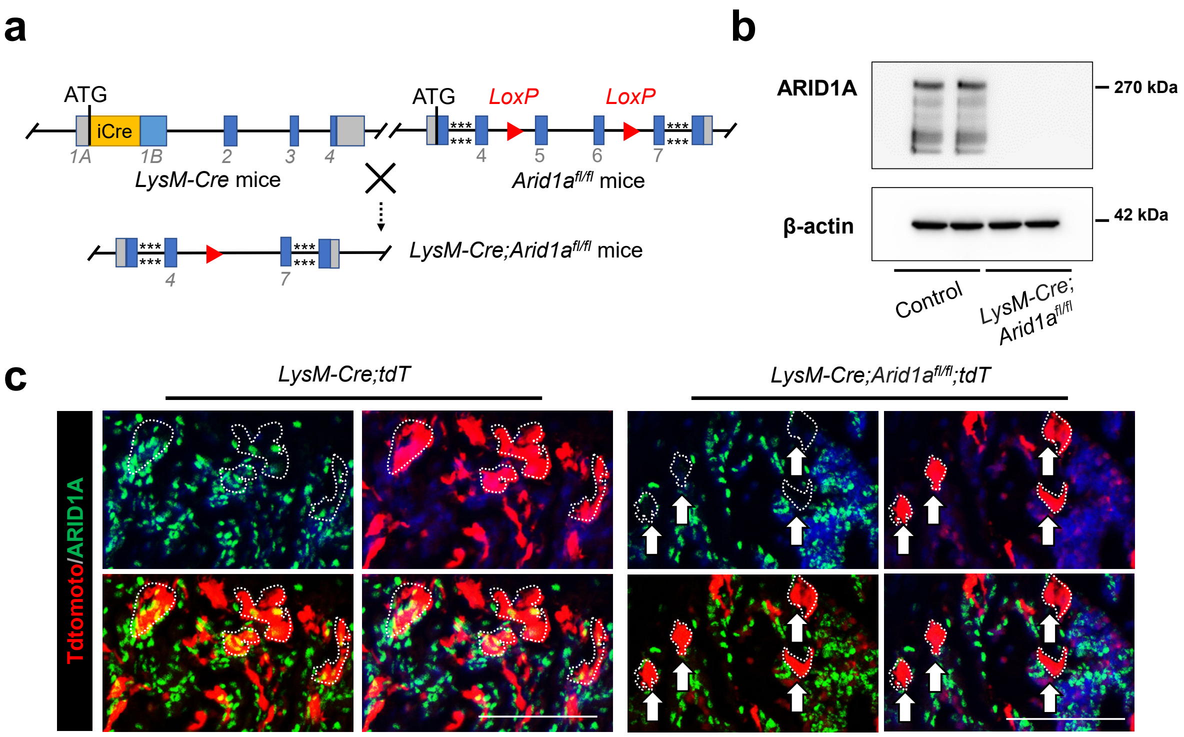

Supplementary Fig. 1 ***Arid1a*** **knockout strategy and efficiency in** ***LysM-Cre;Arid1a$^{fl/fl}$*** **mice. a** Illustration of *Arid1a* deletion in *LysM*-expressing lineage. Mice-bearing *loxP* sites encompassing the *Arid1a exon5-exon6* (*Arid1a$^{fl/fl}$* mice) were crossed with those expressing Cre recombinase driven by the lysozyme M promoter (*LysM-Cre*). **b** ARID1A protein expression in BMCs from 4-week-old male control and *LysM-Cre;Arid1a$^{fl/fl}$* mouse after RANKL induction, as measured by western blot. **c** Immunofluorescence staining of ARID1A (green) in the distal femur of 4-week-old male *LysM-Cre;tdT* mice *and LysM-Cre;Arid1a$^{fl/fl}$;tdT* mice. Arrows indicate ARID1A-LysM+ myeloid lineage. Scale bar, 100 μm. All experiments were performed in triplicates unless otherwise stated. Source data are provided in the Source data file.

Supplementary Fig. 2

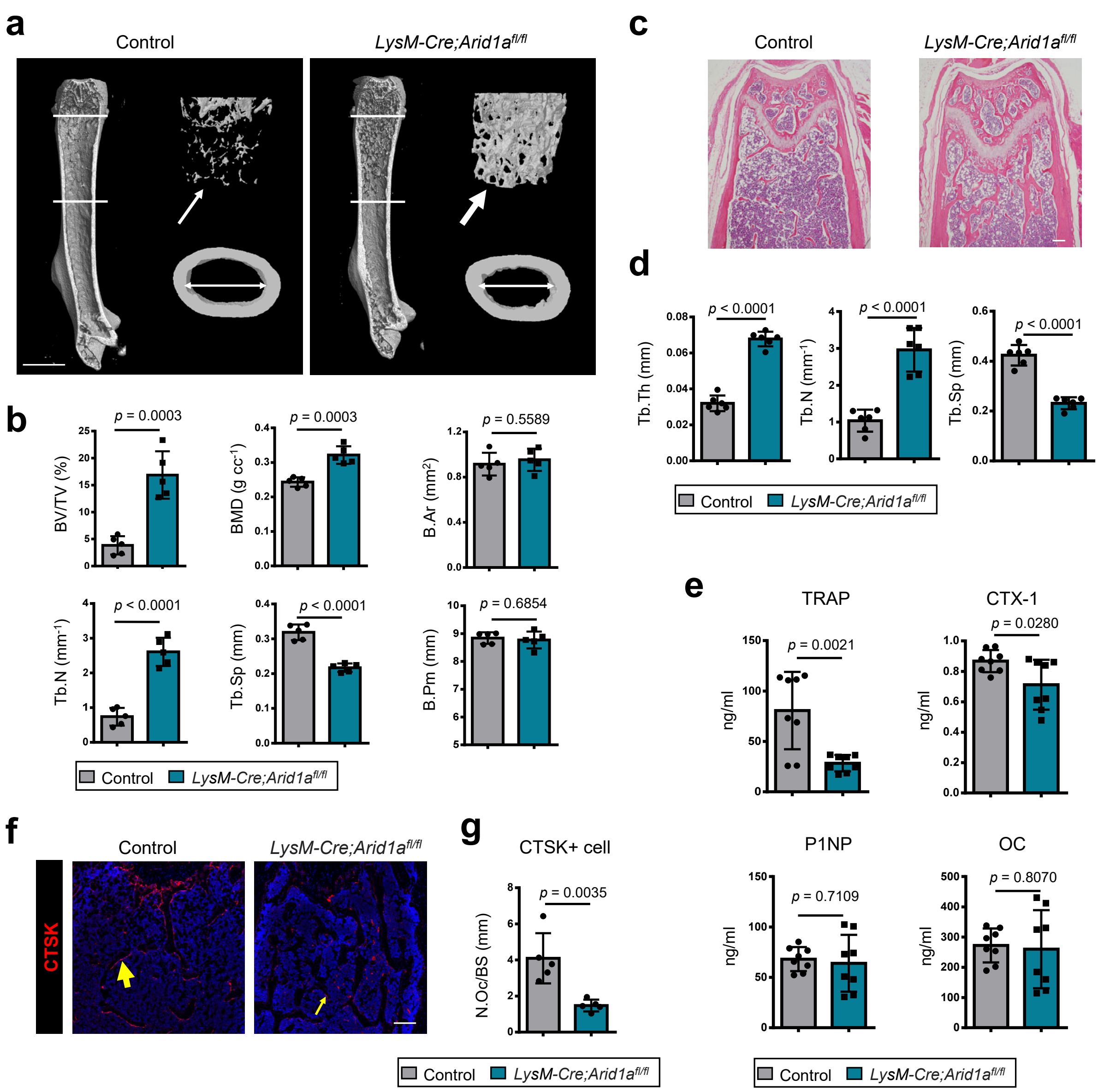

Supplementary Fig. 2. Loss of *Arid1a* in myeloid lineage leads to excessive bone mass. a Representative micro-CT image of the distal trabecular bone and cortical bone of femurs from 6-month-old female *LysM-Cre;Arid1a*$^{fl/fl}$ mice and littermate control mice. Thicker arrows indicate the increased bone mass. Scale bar, 2 mm. **b** Quantification analysis of bone volume/tissue volume ratio (BV/TV), bone mineral density (BMD), trabecular number (Tb.N), and trabecular separation (Tb.Sp) of the distal femoral trabecular bone, mean total crossectional bone area (B. Ar) and mean total

crossectional bone perimeter (B. Pm) of cortical bone in the femoral midshaft from 6-month-old female *LysM-Cre;Arid1a$^{fl/fl}$* mice and littermate control mice. *n* = 5 samples. **c** H&E staining and **d** quantification analysis of trabecular thickness (Tb.Th), trabecular number (Tb.N), and trabecular separation (Tb.Sp) of the distal femoral trabecular bone from 6-month-old female *LysM-Cre;Arid1a$^{fl/fl}$* mice and littermate control mice. Scale bar, 200 μm. *n* = 6 samples. **e** Quantitative measurements of serum biomarkers of bone resorption (TRAP and CTX-I) and bone formation (OCN and P1NP) in 3-month-old male *LysM-Cre;Arid1a$^{fl/fl}$* mice and control mice. *n* = 8 samples. **f** CTSK immunofluorescence (red) staining and **g** quantification analysis of the distal femoral trabecular bone from 6-month-old female *LysM-Cre;Arid1a$^{fl/fl}$* mice and littermate control mice. *n* = 5 samples. Scale bar, 200 μm. Yellow arrows indicate positive signals. All data in this figure are represented as mean ± SD. Two-tailed Student's *t*-test for **b**, **d**, **e** and **g**. All experiments were performed in triplicates unless otherwise stated. Source data and exact *p* values are provided in the Source data file.

Supplementary Fig. 3

a

Cluster	Cell number (proportion)	
	Control	*LysM-Cre;Arid1a*$^{fl/fl}$
0	880 (15.14%)	1223 (23.05%)
1	909 (15.64%)	758 (14.29%)
2	755 (12.99%)	873 (16.46%)
3	1055 (18.15%)	403 (7.6%)
4	942 (16.21%)	302 (5.69%)
5	430 (7.4%)	808 (15.23%)
6	422 (7.26%)	324 (6.11%)
7	299 (5.14%)	289 (5.45%)
8	88 (1.51%)	135 (2.54%)
9	15 (0.26%)	111 (2.09%)
10	18 (0.31%)	79 (1.49%)

d

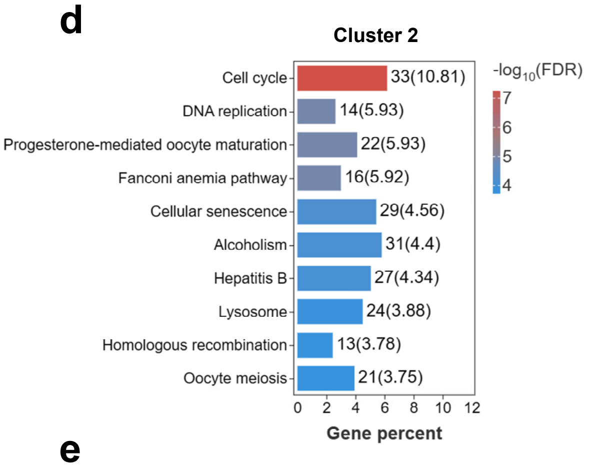

b **c** **e** **f**

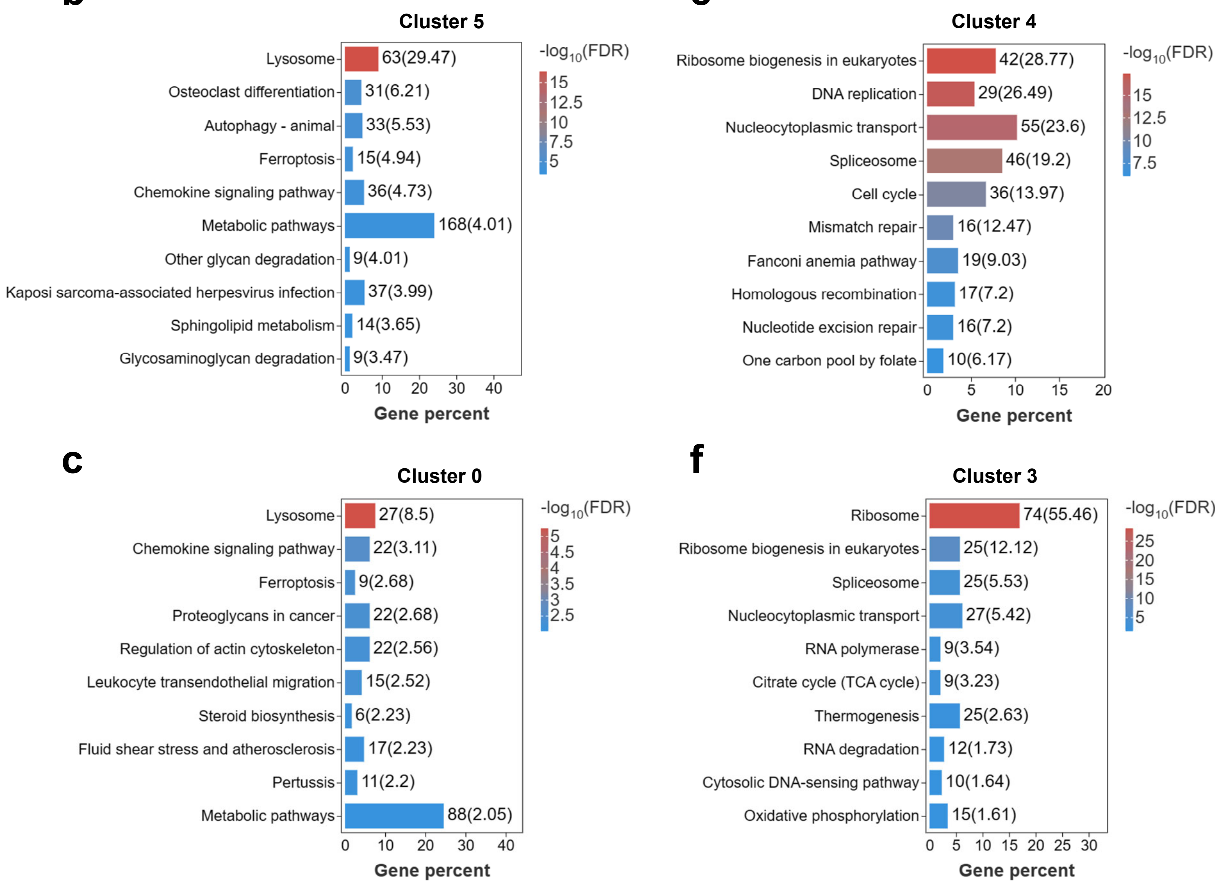

Supplementary Fig. 3 The proportion and KEGG enrichment analysis of each cell cluster based on the scRNA-seq data. a Change in the proportion of each cell cluster between the control group and *LysM-Cre;Arid1a*$^{fl/fl}$ group. **b-f** Bar plot of top 10 KEGG pathway enrichment statistics of differentially expressed genes (DEGs) profiles between cluster 5, 0, 2, 4, and 3 in the control group. Hypergeometric distribution test for **b-f**.The

y-axis represents the name of the pathway and the x-axis represents the gene percent. Color bar indicates the FDR value.

Supplementary Fig. 4

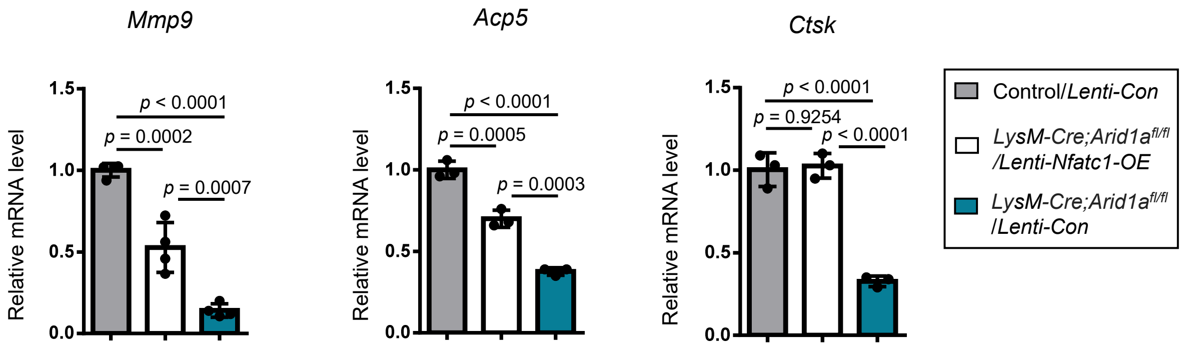

Supplementary Fig. 4 The constituently expressed *Nfatc1* partially rescues the defective OC differentiation after loss of *Arid1a*. The mRNA expression of *Mmp9, Acp5* and *Ctsk* in *Nfatc1*-overexpressed (OE) and control BMCs from 4-week-old male *LysM-Cre;Arid1a*$^{fl/fl}$ mice compared with that from control littermates after RANKL-induction, as measured by qPCR. *n* = 4 biologically independent samples for *Mmp9*. *n* = 3 biologically independent samples for *Acp5* and *Ctsk*. All data in this figure are represented as mean ± SD. one-way analysis of variance (ANOVA) with Tukey's multiple comparisons test. All experiments were performed in triplicates unless otherwise stated. Source data and exact *p* values are provided in the Source data file.

Supplementary Fig. 5

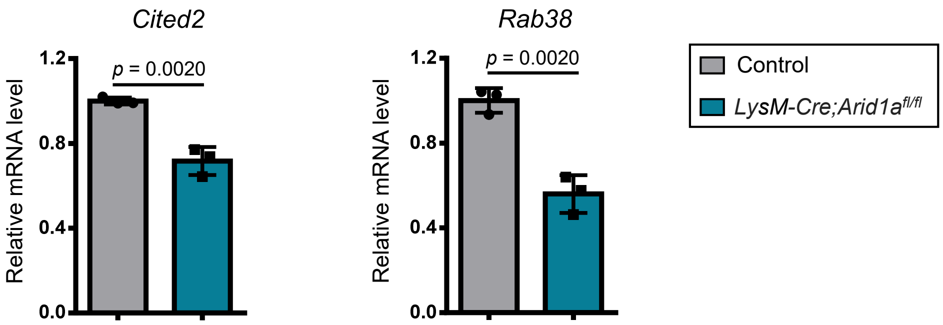

Supplementary Fig. 5 Expression change of fate decision marker *Cited2* and *Rab38* after loss of *Arid1a*. The mRNA expression of Cited2 and *Rab38* in BMCs from 4-week-old male *LysM-Cre;Arid1a*$^{fl/fl}$ mice compared with that from control littermates after RANKL-induction, as measured by qPCR. n = 3 biologically independent samples. All data in this figure are represented as mean ± SD. Two-tailed Student's *t*-test. All experiments were performed in triplicates unless otherwise stated. Source data are provided in the Source data file.

Supplementary Fig. 6

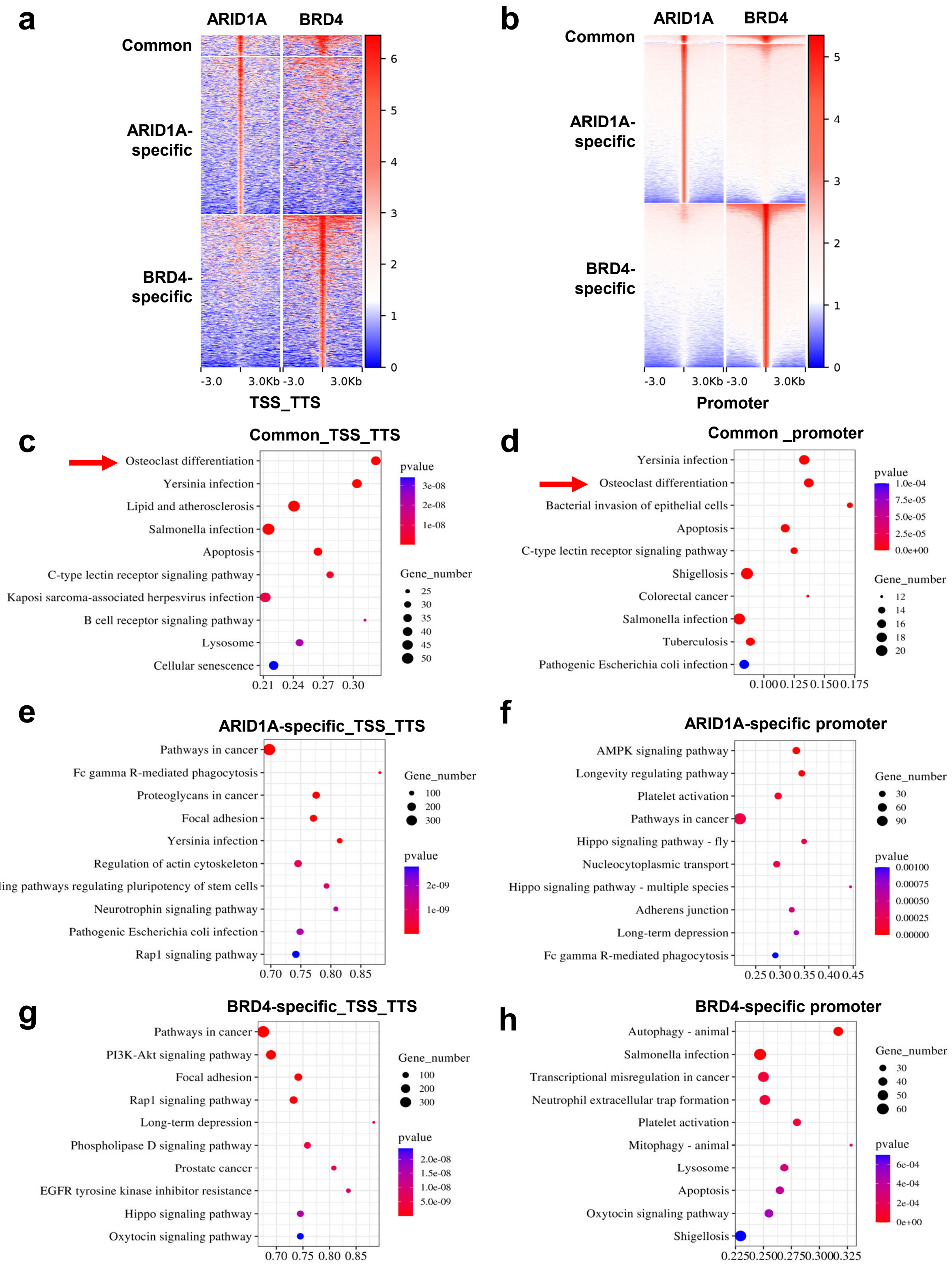

Supplementary Fig. 6 Comparison between the binding profiles of ARID1A and BRD4 during osteoclastogenesis. a, b Heatmap representation of ChIP-seq against ARID1A and BRD4 in ± 3 kb around TSS_TTS (**a)** and promoter **(b)**. **c**-**h** Scatter plot of top 10 KEGG pathway enrichment statistics. Hypergeometric distribution test for **c-h**.The y-axis represents the name of the pathway and the x-axis represents the rich factor. Dot size represents the number of genes and the color bar indicates the p value. Arrow indicates that the enriched pathway of OC differentiation process.

Supplementary Fig. 7

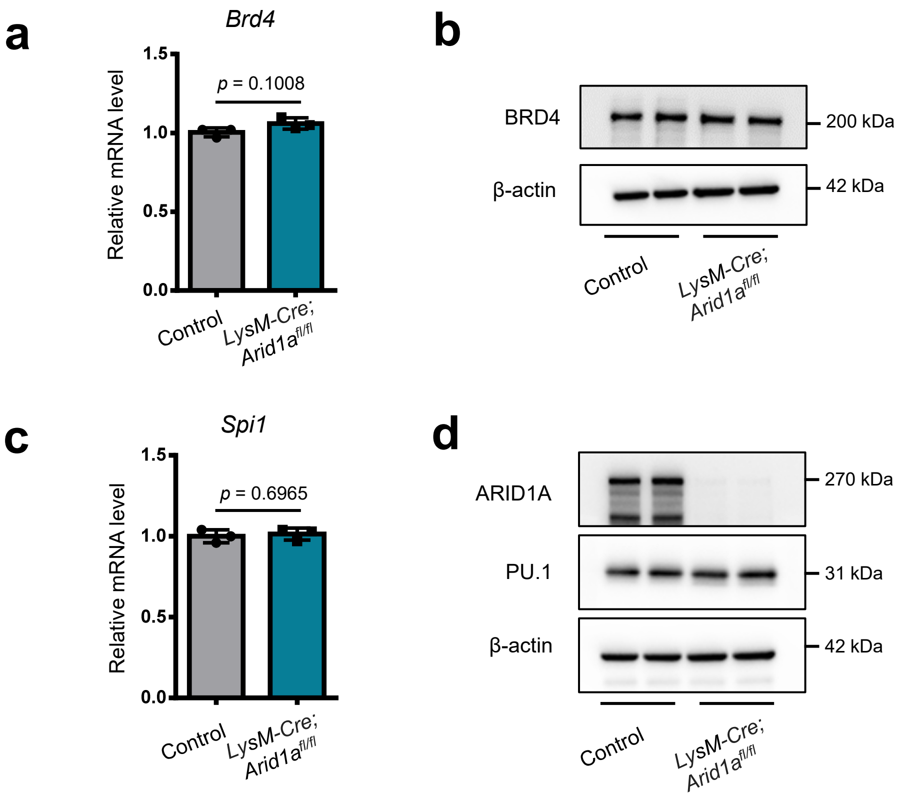

Supplementary Fig. 7 Expression change of BRD4 and PU.1 after loss of *Arid1a*. **a** The mRNA expression of *Brd4* in BMCs from 4-week-old male *LysM-Cre;Arid1a*$^{fl/fl}$ mice compared with that from control littermates after RANKL-induction, as measured by qPCR. *n* = 3 biologically independent samples. **b** The protein expression of BRD4 in BMCs from 4-week-old male *LysM-Cre;Arid1a*$^{fl/fl}$ mice compared with that from control littermates after RANKL-induction, as measured by western blot. **c** The mRNA expression of *Spi1* (*Pu.1*) in BMCs from 4-week-old male *LysM-Cre;Arid1a*$^{fl/fl}$ mice compared with that from control littermates after RANKL-induction, as measured by qPCR. *n* = 3 biologically independent samples. **d** The protein expression of ARID1A and PU.1 in BMCs from 4-week-old male *LysM-Cre;Arid1a*$^{fl/fl}$ mice compared with that from control littermates after RANKL-induction, as measured by western blot. All data in this figure are represented as mean ± SD. Two-tailed Student's *t*-test for **a** and **c**. All experiments were performed in triplicates unless otherwise stated. Source data are provided in the Source data file.

Supplementary Fig. 8

a

Motif logo

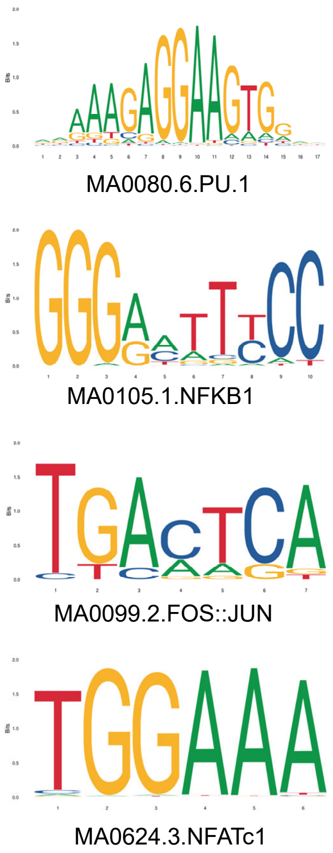

b

	motif_ID	Start	End	Predicted sequence	Relative score
E1	MA0080.6.PU.1	302	318	GCCAGGAGGAAGAGGAG	0.84
		391	407	ATCAAGAGGAACAGGGG	0.84
		424	440	AGGAAAAGGAAGGAGAG	0.83
		308	324	AGGAAGAGGAGGAGCAG	0.82
		320	336	AGCAGGAGGAAGAAGGG	0.82
		336	352	GGGGGGAGGAAATGAAG	0.81
		1	17	AGGAAGAGGAAAGAGGC	0.80
		418	434	ATATAAAGGAAAAGGAA	0.80
	MA0105.1.NFKB1	546	555	GGGAACTCCT	0.83
	MA0099.2.FOS::JUN	231	237	TGAGACA	0.83
		174	180	TTAAACA	0.82
	MA0624.3.NFATc1	7	12	AGGAAA	0.87
		241	246	AGGAAA	0.87
		342	347	AGGAAA	0.87
		381	386	AGGAAA	0.87
		424	429	AGGAAA	0.87
		183	188	GGGAAA	0.86
		265	270	TGAAAA	0.85
		458	463	TGGAAG	0.84
		280	285	TGGAGA	0.84
		357	362	TGGAGA	0.84
		545	550	TGGGAA	0.83
E2	MA0080.6.PU.1	7	23	AGGAAGTGGAAGTTGGT	0.84
		1	17	CCTTTGAGGAAGTGGAA	0.83
	MA0105.1.NFKB1	499	508	TGGATTTTCC	0.85
	MA0099.2.FOS::JUN	79	85	GGACTCA	0.85
		633	639	TGAGTGA	0.83
		519	525	TGAATTA	0.82
	MA0624.3.NFATc1	389	394	TGGACA	0.82
		212	217	AGGAAA	0.87
		13	18	TGGAAG	0.84
E3	MA0080.6.PU.1	63	79	AGAAAGCGGAACTAGTG	0.81
	MA0624.3.NFATc1	58	63	TGTAAA	0.85
		258	263	TGGAAG	0.84

Supplementary Fig. 8 The motif location of PU.1, NFKB1, FOS::JUN and NFATc1 in the *Nfatc1 SE* region. a The motif logo and **b** motif hits of PU.1, NFKB1, FOS::JUN and NFATc1 in the enhancer region 1 (*E1*, 0.59kb, Chr18:80,758,246-80,758,838), enhancer region 2 (*E2*, 0.71kb, Chr18:80,745,769-80,746,481) and enhancer region 3 (*E3*, 0.34kb, Chr18:80,734,924-80,735,265), predicted by JASPAR.

Supplementary Fig. 9

a

Rank	GO. ID	Gene set_ Up in *LysM-Cre;Arid1a$^{fl/fl}$* group	NES	NOM p-val
1	GO_0035456	RESPONSE TO INTERFERON-BETA	2.36	0.000
2	GO_0035458	CELLULAR RESPONSE TO INTERFERON-BETA	2.35	0.000
3	GO_0016503	PHEROMONE RECEPTOR ACTIVITY	2.27	0.000
4	GO_0048525	NEGATIVE REGULATION OF VIRAL PROCESS	2.25	0.000
5	GO_0045071	NEGATIVE REGULATION OF VIRAL GENOME REPLICATION	2.15	0.000
6	GO_0048002	ANTIGEN PROCESSING AND PRESENTATION OF PEPTIDE ANTIGEN	2.12	0.000
7	GO_0009620	RESPONSE TO FUNGUS	2.11	0.000
8	GO_0051673	MEMBRANE DISRUPTION IN ANOTHER ORGANISM	2.11	0.000
9	GO_0002483	ANTIGEN PROCESSING AND PRESENTATION OF ENDOGENOUS PEPTIDE ANTIGEN	2.10	0.000
10	GO_0140546	DEFENSE RESPONSE TO SYMBIONT	2.10	0.000

b

Rank	GO. ID	Gene set_ Down in *LysM-Cre;Arid1a$^{fl/fl}$* group	NES	NOM p-val
1	GO_0003735	STRUCTURAL CONSTITUENT OF RIBOSOME	-2.41	0.000
2	GO_0044391	RIBOSOMAL SUBUNIT	-2.37	0.000
3	GO_0022626	CYTOSOLIC RIBOSOME	-2.37	0.000
4	GO_0042254	RIBOSOME BIOGENESIS	-2.36	0.000
5	GO_0022613	RIBONUCLEOPROTEIN COMPLEX BIOGENESIS	-2.30	0.000
6	GO_0098798	MITOCHONDRIAL PROTEIN-CONTAINING COMPLEX	-2.29	0.000
7	GO_0002181	CYTOPLASMIC TRANSLATION	-2.29	0.000
8	GO_0006364	RRNA PROCESSING	-2.26	0.000
9	GO_0005840	ANTIGEN PROCESSING AND RIBOSOME	-2.26	0.000
10	GO_0022625	CYTOSOLIC LARGE RIBOSOMAL SUBUNIT	-2.25	0.000

c

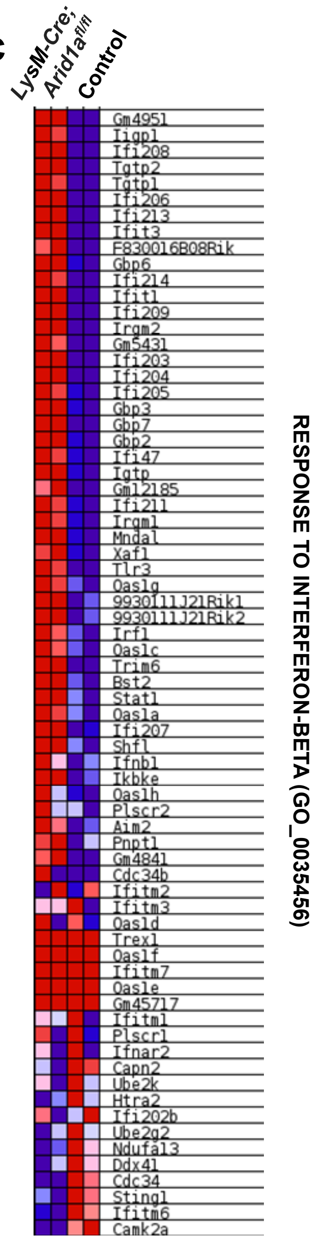

Supplementary Fig. 9 Gene set enrichment analysis (GSEA) analysis of DEGs in *LysM-Cre;Arid1a$^{fl/fl}$* group compared with control group based on bulk RNA-seq. GSEA analysis of the top 10 upregulated gene sets **a** and downregulated gene sets **b** in *LysM-Cre;Arid1a$^{fl/fl}$* group compared with the control group. **c** Heatmap hierarchical clustering of GO term_0035456 in *LysM-Cre;Arid1a$^{fl/fl}$* group compared with control

group. *n* = 2 biologically independent samples. Empirical phenotype-based permutation test for **a** and **b**.

Supplementary Fig. 10

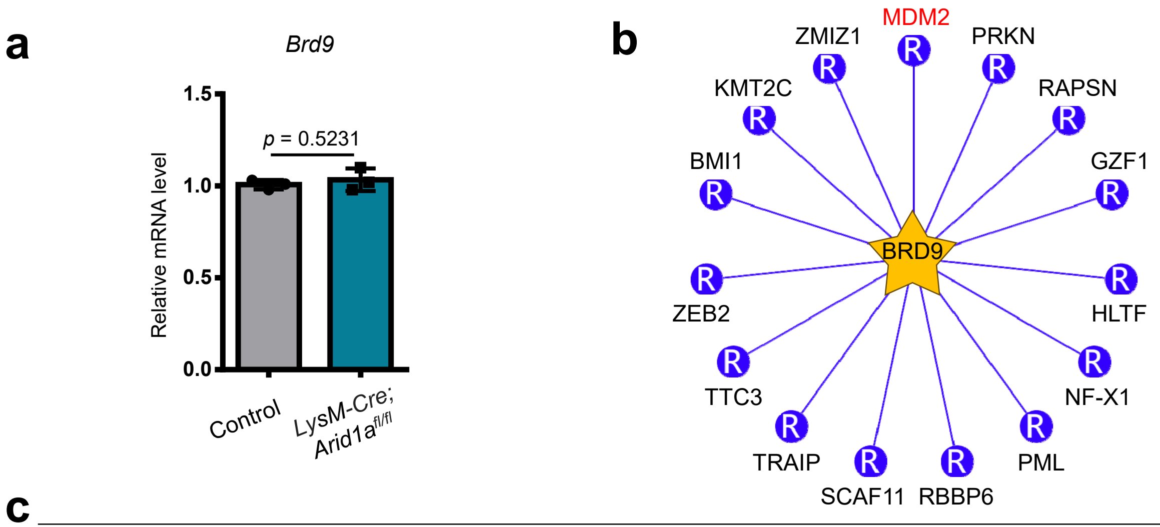

c

Gene symbol	Gene description	Confidence score	Confidence level	*LysM-Cre;Arid1a*$^{fl/fl}$ /control	
				**Log2 Fold Change**	**P value**
Mdm2	E3 ubiquitin-protein ligase Mdm2	0.874	HIGH	-0.581952782	5.20E-08
Prkn	E3 ubiquitin-protein ligase parkin	0.845	HIGH	0.842641884	0.307277063
Rapsn	43 kDa receptor-associated protein of the synapse	0.845	HIGH	-2.845657722	0.351642069
Gzf1	GDNF-inducible zinc finger protein 1	0.809	HIGH	-0.281077415	0.091868429
Hltf	Helicase-like transcription factor	0.809	HIGH	0.373170206	0.00600114
Nfx1	Transcriptional repressor NF-X1	0.809	HIGH	-0.239733155	0.132201465
Pml	Protein PML	0.809	HIGH	0.950631487	7.18E-16
Rbbp6	E3 ubiquitin-protein ligase RBBP6	0.809	HIGH	-0.013502365	0.924569292
Scaf11	-	0.809	HIGH	0.043611878	0.710527254
Traip	E3 ubiquitin-protein ligase TRAIP	0.809	HIGH	-0.249363218	0.147512453
Ttc3	E3 ubiquitin-protein ligase TTC3	0.809	HIGH	0.154448965	0.337877298
Zeb2	Zinc finger E-box-binding homeobox 2	0.809	HIGH	0.119787147	0.553950008
Bmi1	Polycomb complex protein BMI-1	0.804	HIGH	-0.182417261	0.203998358
Kmt2c	Histone-lysine N-methyltransferase 2C	0.803	HIGH	0.374197176	0.1838757
Zmiz1	Zinc finger MIZ domain-containing protein 1	0.803	HIGH	0.007336045	0.953592848

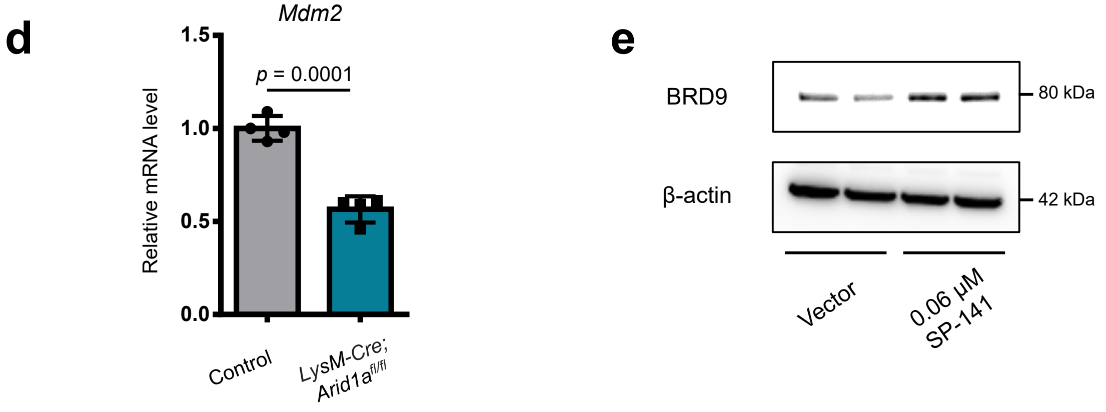

Supplementary Fig. 10 Change of BRD9 in RANKL-induced BMCs after loss of *Arid1a*. a The mRNA expression of *Brd9* in BMCs from 4-week-old male *LysM-*

Cre;Arid1a$^{fl/fl}$ mice compared with that from control littermates, as measured by qPCR. *n* = 3 biologically independent samples. **b** The predicted potential E3 ligases/deubiquitinases (DUBs) for BRD9 using UbiBrowser 2.0 software. **c** The mRNA expression change of top 15 predicted ubiquitin ligases/DUBs in BMDMs from control and *LysM-Cre;Arid1a$^{fl/fl}$* mouse. **d** The mRNA expression of *Mdm2* in BMCs from *LysM-Cre;Arid1a$^{fl/fl}$* mice compared with that from control littermates, as measured by qPCR. *n* = 4 biologically independent samples. **e** The protein expression of BRD9 in 0.06 μM SP-141 (MDM2 inhibitor) or control vector treated BMCs after RANKL-induction, as measured by western blot. Two-tailed Student's *t*-test for **a** and **d**. Negative binomial distribution used for **c**. All experiments were performed in triplicates unless otherwise stated. Source data are provided in the Source data file.
