# Supplementary material for: ARID1A safeguards the canalization of the cell fate decision during osteoclastogenesis
Source: Nat Commun. 2024 Jul 17;15:5994. doi: 10.1038/s41467-024-50225-z (PMC11252270; doi:10.1038/s41467-024-50225-z)
Supplement: Supplementary file 3 — Description of Additional Supplementary Files [file 41467_2024_50225_MOESM3_ESM.pdf]

<u>Description of Additional Supplementary Files</u>

Supplementary Data Legends:

Supplementary Data 1. The 728 genes associated with SEs identified by H3K27Ac ChIP-Seq.

Supplementary Data 2. The 143 genes common in ARID1A occupied and SE-associated gene list.

Supplementary Data 3. The 44 OC differentiation-associated differentiated expressed genes after loss of ARID1A within cell cluster 4.

Supplementary Data 4. List of primer sequences used for q-PCR.

Supplementary Data 5. List of 3C-qPCR primers.
