# Supplementary material for: ARID1A safeguards the canalization of the cell fate decision during osteoclastogenesis
Source: Nat Commun. 2024 Jul 17;15:5994. doi: 10.1038/s41467-024-50225-z (PMC11252270; doi:10.1038/s41467-024-50225-z)
Supplement: Supplementary file 9 — Reporting Summary [file 41467_2024_50225_MOESM9_ESM.pdf]

nature portfolio

Corresponding author(s): Xinquan Jiang, Guangzheng Yang

Last updated by author(s): Jun 23, 2024

Reporting Summary

Nature Portfolio wishes to improve the reproducibility of the work that we publish. This form provides structure for consistency and transparency in reporting. For further information on Nature Portfolio policies, see our Editorial Policies and the Editorial Policy Checklist.

Statistics

For all statistical analyses, confirm that the following items are present in the figure legend, table legend, main text, or Methods section.

n/a	Confirmed	
☐	☒	The exact sample size (*n*) for each experimental group/condition, given as a discrete number and unit of measurement
☐	☒	A statement on whether measurements were taken from distinct samples or whether the same sample was measured repeatedly
☐	☒	The statistical test(s) used AND whether they are one- or two-sided<br>*Only common tests should be described solely by name; describe more complex techniques in the Methods section.*
☒	☐	A description of all covariates tested
☐	☒	A description of any assumptions or corrections, such as tests of normality and adjustment for multiple comparisons
☐	☒	A full description of the statistical parameters including central tendency (e.g. means) or other basic estimates (e.g. regression coefficient) AND variation (e.g. standard deviation) or associated estimates of uncertainty (e.g. confidence intervals)
☐	☒	For null hypothesis testing, the test statistic (e.g. *F*, *t*, *r*) with confidence intervals, effect sizes, degrees of freedom and *P* value noted<br>*Give P values as exact values whenever suitable.*
☒	☐	For Bayesian analysis, information on the choice of priors and Markov chain Monte Carlo settings
☒	☐	For hierarchical and complex designs, identification of the appropriate level for tests and full reporting of outcomes
☒	☐	Estimates of effect sizes (e.g. Cohen's *d*, Pearson's *r*), indicating how they were calculated

Our web collection on statistics for biologists contains articles on many of the points above.

Software and code

Policy information about availability of computer code

Data collection

NIS-Elements software (v4.5000.1117.0) and DP2-TWAIN software (v3.0.0.6212) were used to acquire images. Western blots were detected using UVITEC Alliance system (v16.0.3.0). Real-time reverse transcription PCR was performed using Roche LightCycler 480 system (v1.5.1.74). RNA-seq and ChIP-seq were conducted on Illumina NovaSeq 6000 platform.

Data analysis

CT data were reconstructed with NRecon software (v1.7.1.0, Bruker, Kontich, Belgium), and analyzed using the program CTAn (v1.16, Bruker, Kontich, Belgium).
Statistical analyses were performed with GraphPad Prism v6.01 software.
Single-cell RNA-sequencing analysis:Single cells were loaded on a 10X Genomics GemCode Single-cell instrument. Libraries were generated and sequenced from the cDNAs with Chromium Next GEM Single Cell 3′ Reagent Kits v3.1. and subjected to Illumina NovaSeq 6000. Quality control, alignment, quantification, and aggregation of sample count matrices were performed using the 10x Genomics Cell Ranger pipeline according to the manufacturer's protocol. Significantly upregulated genes were identified with at least 1.28-fold overexpressed in the target cluster, expressed in more than 25% of the cells belonging to the target cluster and the p-value is less than 0.01. The differentially expressed genes in the given cluster were further conducted with KEGG pathway enrichment analysis. seudotime analysis was conducted using monocle2, a method that can predict the differentiation trajectory based on an individual cell's asynchronous progression of the process with a standard protocol with default parameters. The estimation of the root of the trajectory was based on the cluster identities and marker gene analysis. Partition-based graph abstraction (PAGA), which allows robust reconstruction branching gene expression changes across different datasets, was also conducted according to standard protocol. Correlation statistics and significance calculations for each gene were conducted using the Rfast2 software package. Then the significantly differential expression genes with FDR <1e-7 and genes with similar trends in expression were identified on the pseudotime axis.
RNA-sequencing analysis: libraries were prepared using the NEBNext Ultra II RNA Library Prep Kit and then sequenced on the Illumina NovaSeq 6000 platform. Raw reads were filtered using Cutadapt (v1.15) and aligned with the GRCm39 genome using HISAT2 v2.0.5. Read

Count values on each gene were compared using HTSeq (v0.9.1) and normalized to FPKM. Then difference expression of genes was analyzed using DESeq (v1.30.0) (fold change >= 2 and p < 0.05). GSEA analysis was performed to functionally annotate the relevant genes and assess the enriched signaling pathways using GSEA_Linux_4.1.0.
ChIP-seq: Raw reads were filtered using FASTX-Toolkit (v0.0.14) (http://hannonlab.cshl.edu/fastx_toolkit/). Clean reads were aligned to the GRCm39 mouse genome using Bowtie 2 (v2.3.5.1). Multiple aligned reads were filtered out using Picard (v2.27.5) (http://broadinstitute.github.io/picard/). Then the unique mapped reads without duplicated reads were called for peaks using MACS2 (v2.2.71) with default parameters. The peaks were annotated using ChIPseeker (v1.20.0). For motif enrichment analysis we used AME with the same background model and negative control. AME tool of the MEME suite provides motifs that are enriched compared to the shuffled background. We set the E-value threshold (E-value<=10, Fisher's exact test) for reporting enriched motifs. We used motif database JASPAR2022_CORE_vertebrates_non-redundant_v2. meme to test for enrichment.

For manuscripts utilizing custom algorithms or software that are central to the research but not yet described in published literature, software must be made available to editors and reviewers. We strongly encourage code deposition in a community repository (e.g. GitHub). See the Nature Portfolio guidelines for submitting code & software for further information.

Data

Policy information about availability of data

All manuscripts must include a data availability statement. This statement should provide the following information, where applicable:

- Accession codes, unique identifiers, or web links for publicly available datasets
- A description of any restrictions on data availability
- For clinical datasets or third party data, please ensure that the statement adheres to our policy

The scRNA, bulk mRNA, and ChIP-sequencing data generated in this study have been deposited in the Gene Expression Omnibus database under accession code GSE245258 [https://www.ncbi.nlm.nih.gov/geo/query/acc.cgi?acc=GSE245258]. GRCm39 genome is referenced in this study [http://asia.ensembl.org/Mus_musculus/Info/Index]. The other relevant data generated in this study are provided in the Supplementary Information/Source Data file.

Research involving human participants, their data, or biological material

Policy information about studies with human participants or human data. See also policy information about sex, gender (identity/presentation), and sexual orientation and race, ethnicity and racism.

Reporting on sex and gender	N/A
Reporting on race, ethnicity, or other socially relevant groupings	N/A
Population characteristics	N/A
Recruitment	N/A
Ethics oversight	N/A

Note that full information on the approval of the study protocol must also be provided in the manuscript.

Field-specific reporting

Please select the one below that is the best fit for your research. If you are not sure, read the appropriate sections before making your selection.

☒ Life sciences ☐ Behavioural & social sciences ☐ Ecological, evolutionary & environmental sciences

For a reference copy of the document with all sections, see nature.com/documents/nr-reporting-summary-flat.pdf

Life sciences study design

All studies must disclose on these points even when the disclosure is negative.

Sample size	No statistical methods were used to predetermine sample size. At least n = 3 mice of each groups were analyzed with exact sample size declared in corresponding figure legends. This was shown to be sufficient in previous studies, e.g. Deng, C. et al . Nat Commun 12, 2174 (2021); Sun, W. et al. Nat Commun 13, 2899 (2022); Jacome-Galarza, C.E. et al. Nature 568, 541–545 (2019), to discern statistically significant differences. In molecular biology experiments, n = 3 was chosen to generate p-values to determine if results are significant.
Data exclusions	No data were excluded in this study.
Replication	The in vitro experiments reported in the manuscript were replicated independently at least three times to confirm reproducibility. The exact sample size in terms of mice in all experiments are declared in corresponding figure legends.
Randomization	Samples and mice were randomly allocated to different groups.

Blinding | Analyses were done objectively. Blinding was not relevant for this study.

Reporting for specific materials, systems and methods

We require information from authors about some types of materials, experimental systems and methods used in many studies. Here, indicate whether each material, system or method listed is relevant to your study. If you are not sure if a list item applies to your research, read the appropriate section before selecting a response.

Materials & experimental systems

n/a	Involved in the study
☐	☒ Antibodies
☐	☒ Eukaryotic cell lines
☒	☐ Palaeontology and archaeology
☐	☒ Animals and other organisms
☒	☐ Clinical data
☒	☐ Dual use research of concern
☒	☐ Plants

Methods

n/a	Involved in the study
☐	☒ ChIP-seq
☒	☐ Flow cytometry
☒	☐ MRI-based neuroimaging

Antibodies

Antibodies used

Antibodies used in immunofluorescence staining are as following: ARID1A antibody (Cell signaling, 12354, 1:100; novusbio, NBP2-61623, 1:100), CTSK antibody (Abcam, ab37259, 1:100), ACP5 antibody (Abcam, ab235448, 1:100), NFATc1 antibody (Bioworld, bs6677, 1:100), BRD4 antibody (Bethyl Laboratories, A700-004, 1:100), PU.1 antibody (abnova, H00006688-M02, 1:100), STAT1 antibody (Cell signaling, 9172, 1:100), goat anti-rabbit IgG (H+L) cross-adsorbed secondary antibody, alexa fluor 488 (Thermo Fisher Scientific, A-11008, 1:200), donkey anti-rabbit IgG (H+L) highly cross-adsorbed secondary antibody, alexa fluor 594 (Thermo Fisher Scientific, A-21207, 1:200), goat anti-mouse IgG (H+L) cross-adsorbed secondary antibody, alexa fluor 488 (Thermo Fisher Scientific, A-11001, 1:200), goat anti-mouse IgG (H+L) cross-adsorbed secondary antibody, alexa fluor 594 (Thermo Fisher Scientific, A-11005, 1:200), goat anti-mouse IgG (H+L) cross-adsorbed secondary antibody, alexa fluor™ 647 (Thermo Fisher Scientific, A-21235, 1:200).

Antibodies used in western blot as following: ARID1A antibody (Cell signaling, 12354, 1:1000), MMP9 antibody (Abcam, ab228402, 1:1000), CTSK antibody (Abcam, ab37259, 1:1000), NFATc1 (BioLegend, 649601, 1:500), BRD4 antibody (Bethyl Laboratories, A700-004, 1:1000), PU.1 antibody (Abcam, ab227835, 1:1000), BRD9 antibody (Abcam, ab259839, 1:1000), STAT1 antibody (Cell signaling, 9172, 1:1000), β-actin antibody (Abcam, ab20272HRP, 1:5000), mouse IgG HRP-conjugated antibody (R&D, HAF007, 1:1000) and rabbit IgG HRP-conjugated antibody (R&D, HAF008, 1:1000).

ARID1A antibody (Cell signaling, 12354, 1:100), BRD9 antibody (Bethyl Laboratories, A700-153, 1:100) or normal Rabbit IgG (Cell Signaling, 2729, the same concentration as the specific target antibody) were used for immunoprecipitation. Immune complexes were subjected to immunoblotting with ARID1A antibody (Cell signaling, 12354, 1:1000), BRD4 antibody (Bethyl Laboratories, A700-004, 1:1000), PU.1 antibody (Abcam, ab227835, 1:1000), BRD9 antibody (Abcam, ab259839, 1:1000).

H3K27Ac antibody (Cell signaling, 8173, 1:100), ARID1A antibody (Cell signaling, 12354, 1:100), BRD4 antibody (Bethyl Laboratories, A700-004, 1:100), PU.1 antibody (Abcam, ab227835, 1:100) with normal rabbit IgG (Cell Signaling, 2729, the same concentration as the specific target antibody) as a non-specific IgG control were used for chromatin immunoprecipitation.

Validation

All antibodies were commercial available and characterized by manufacturers online.
1. ARID1A antibody (https://www.cellsignal.com/products/primary-antibodies/arid1a-baf250a-d2a8u-rabbit-mab/12354);
2. ARID1A antibody (https://www.novusbio.com/products/arid1a-antibody-cl3595_nbp2-61623);
2. CTSK antibody (https://www.abcam.com/cathepsin-k-antibody-3f9-ab37259.html);
3. ACP5 antibody (https://www.abcam.cn/acid-phosphatase-antibody-epr21787-ab235448.html);
4. NFATc1 antibody (https://bioworlde.com/Primary-Antibodies/149784.html);
5. BRD4 antibody (https://www.biomol.com/products/antibodies/primary-antibodies/general/anti-brd4-recombinant-monoclonal-a700-004-t)
6. PU.1 antibody (https://www.abnova.com/en-global/product/detail/H00006688-M02)
7. STAT1 antibody (https://www.cellsignal.com/products/primary-antibodies/stat1-antibody/9172);
8. Goat anti-Rabbit IgG (H+L) Cross-Adsorbed Secondary Antibody, Alexa Fluor 488 (https://www.thermofisher.com/antibody/product/Goat-anti-Rabbit-IgG-H-L-Cross-Adsorbed-Secondary-Antibody-Polyclonal/A-11008);
9. Donkey anti-Rabbit IgG (H+L) Highly Cross-Adsorbed Secondary Antibody, Alexa Fluor™ 594 (https://www.thermofisher.com/antibody/product/Donkey-anti-Rabbit-IgG-H-L-Highly-Cross-Adsorbed-Secondary-Antibody-Polyclonal/A-21207);
10. Goat anti-Mouse IgG (H+L) Cross-Adsorbed Secondary Antibody, Alexa Fluor 488 (https://www.thermofisher.com/antibody/product/Goat-anti-Mouse-IgG-H-L-Cross-Adsorbed-Secondary-Antibody-Polyclonal/A-11001);
11. Goat anti-Mouse IgG (H+L) Cross-Adsorbed Secondary Antibody, Alexa Fluor 594 (https://www.thermofisher.com/antibody/product/Goat-anti-Mouse-IgG-H-L-Cross-Adsorbed-Secondary-Antibody-Polyclonal/A-11005);
12. goat anti-mouse IgG (H+L) cross-adsorbed secondary antibody, alexa fluor™ 647 (https://www.thermofisher.cn/cn/zh/antibody/product/Goat-anti-Mouse-IgG-H-L-Cross-Adsorbed-Secondary-Antibody-Polyclonal/A-21235);
13. MMP9 antibody (https://www.abcam.com/mmp9-antibody-epr22140-154-ab228402.html);
14. NFATc1 antibody (https://www.biolegend.com/en-gb/products/purified-anti-nfatc1-antibody-6993?GroupID=BLG9157);
15. PU.1 antibody (https://www.abcam.com/products/primary-antibodies/pu1spi1-antibody-epr22624-20-chip-grade-ab227835.html);

16. BRD9 antibody (https://www.abcam.cn/brd9-antibody-epr23888-5-ab259839.html);
17. β-actin antibody (https://www.abcam.com/hrp-beta-actin-antibody-mabcam-8226-loading-control-ab20272.html);
18. Mouse IgG HRP-conjugated antibody (https://www.rndsystems.com/cn/products/mouse-igg-hrp-conjugated-antibody_haf007);
19. Rabbit IgG HRP-conjugated antibody (https://www.rndsystems.com/cn/products/rabbit-igg-hrp-conjugated-antibody_haf008);
20. BRD9 antibody (https://www.biomol.com/products/antibodies/primary-antibodies/general/anti-brd9-recombinant-monoclonal-a700-153-t?fs=1236343819).
21. Normal-rabbit-igg antibody (https://www.cellsignal.com/products/primary-antibodies/normal-rabbit-igg/2729);
22. H3K27Ac antibody (https://www.cellsignal.com/products/primary-antibodies/acetyl-histone-h3-lys27-d5e4-xp-rabbit-mab/8173).

Eukaryotic cell lines

Policy information about cell lines and Sex and Gender in Research

Cell line source(s)	RAW264.7 cell line was ordered from the cell bank of Cyagen Biosciences.
Authentication	The cell line has been validated using the short tandem repeat (STR) profiling method.
Mycoplasma contamination	The cell line has been tested negative for mycoplasma contamination.
Commonly misidentified lines (See ICLAC register)	No commonly misidentified cell lines were used.

Animals and other research organisms

Policy information about studies involving animals; ARRIVE guidelines recommended for reporting animal research, and Sex and Gender in Research

Laboratory animals	LysM-Cre mice (Strain NO. T003822), Arid1a-flox mice (Strain NO. T013487) Brd9-flox mice (Strain NO. T008489), and WT C57BL/6J mice (Strain NO. N000013) and tdTomato (Strain NO. 007909) mouse line were used and cross-bred in this study. All mice were used for analysis regardless of sex. All mice were housed in pathogen-free conditions with constant ambient temperature (22 ± 2 °C) and humidity (55 ± 10%), with an alternating 12-hour light/dark cycle. The ages for strains of laboratory animals are between 3-week-old to 6-month-old, and the exact ages of laboratory animals used in each experiment are listed in the figure legend accordingly.
Wild animals	Our study did not involve wild animals.
Reporting on sex	All mice were used for analysis regardless of sex.
Field-collected samples	Our study did not involve samples collected from the field.
Ethics oversight	All animal studies were approved by the Institutional Animal Care and Use Committee at Ninth People's Hospital, School of Medicine, Shanghai Jiao Tong University (SH9H-2022-A926-1).

Note that full information on the approval of the study protocol must also be provided in the manuscript.

Plants

Seed stocks	N/A
Novel plant genotypes	N/A
Authentication	N/A

ChIP-seq

Data deposition

☒ Confirm that both raw and final processed data have been deposited in a public database such as GEO.

☒ Confirm that you have deposited or provided access to graph files (e.g. BED files) for the called peaks.

Data access links *May remain private before publication.*	https://www.ncbi.nlm.nih.gov/geo/query/acc.cgi?acc=GSE245258
Files in database submission	GSM7839482 1_Arid1a

Files in database submission

GSM7839483 1_Input
GSM7839484 C_Brd4
GSM7839485 C_H3K27ac
GSM7839486 C_Input
GSM7839487 M_Brd4
GSM7839488 M_Input
GSM8241279 BMDMs, M_Arid1a
GSM8241280 BMDMs, M_Brd4
GSM8241281 BMDMs, M_Input
GSM8241282 BMDMs, MR_Pu1_2
GSM8241283 BMDMs, MR_Input

Genome browser session (e.g. UCSC)

No longer applicable.

Methodology

Replicates

ChIP-seq experiments were performed in singlicate per condition per experiment.

Sequencing depth

Double-end sequencing was used with 150 bp read length.
GSM7839482 1_Arid1a total reads 93541358, Uniquely aligned reads 64103964
GSM7839483 1_Input total reads 55000802, Uniquely aligned reads 36496672
GSM7839484 C_Brd4 total reads 104836790, Uniquely aligned reads 63399319
GSM7839485 C_H3K27ac total reads 64951172, Uniquely aligned reads 47944961
GSM7839486 C_Input total reads 88046260, Uniquely aligned reads 43695193
GSM7839487 M_Brd4 total reads 113220496, Uniquely aligned reads 56984253
GSM7839488 M_Input total reads 61827020, Uniquely aligned reads 34626259
GSM8241279 BMDMs, M_Arid1a total reads 71609510, Uniquely aligned reads 33542594
GSM8241280 BMDMs, M_Brd4 total reads 67130308, Uniquely aligned reads 50529960
GSM8241281 BMDMs, M_Input total reads 37198094, Uniquely aligned reads 22569934
GSM8241282 BMDMs, MR_Pu1_2 total reads 62843328, Uniquely aligned reads 47951068
GSM8241283 BMDMs, MR_Input total reads 46548128, Uniquely aligned reads 29871114

Antibodies

H3K27Ac antibody (Cell signaling, 8173, 1:100), ARID1A antibody (Cell signaling, 12354, 1:100), BRD4 antibody (Bethyl Laboratories, A700-004, 1:100), PU.1 antibody (Abcam, ab227835, 1:100)

Peak calling parameters

Clean reads were aligned to the GRCm39 mouse genome using Bowtie 2 (v2.3.5.1). Multiple aligned reads were filtered out using Picard (v2.27.5) (http://broadinstitute.github.io/picard/). Then the unique mapped reads without duplicated reads were called for peaks using MACS2 (v2.2.71) with default parameters and $p < 0.05$. The peaks were annotated using ChIPseeker (v1.20.0).

Data quality

Raw reads were filtered using FASTX-Toolkit (v0.0.14) (http://hannonlab.cshl.edu/fastx_toolkit/).p-value < 0.05.
GSM7839482 1_Arid1a call peaks 56564, p-value < 0.05.
GSM7839484 C_Brd4 call peaks 58199, p-value < 0.05.
GSM7839485 C_H3K27ac call peaks 44727, p-value < 0.05.
GSM7839487 M_Brd4 call peaks 256457, p-value < 0.05.
GSM8241279 BMDMs, M_Arid1a call peaks 682855, p-value < 0.05.
GSM8241280 BMDMs, M_Brd4 call peaks 405593, p-value < 0.05.
GSM8241282 BMDMs, MR_Pu1_2 call peaks 413304, p-value < 0.05.

Software

FASTX-Toolkit (v0.0.14), Bowtie 2 (v2.3.5.1), Picard (v2.27.5), MACS2 (v2.2.71), ChIPseeker (v1.20.0)
